# An Account of the Practice of One of the Physicians of the Westminster General Dispensary, and of the Western Dispensary, from the 20th of August, to the 20th of September, 1807

**Published:** 1807-10

**Authors:** Samuel Fothergill

**Affiliations:** Southampton Street, Strand


*An Account of the Practice of one of the Physicians of the Westminster General Dispensary, and of the Western Dispensary, from the 20th of August, to the 20th of September,* 1807.

| ACUTE DISEASES. | | CHRONIC DISEASES. | |
|---|---|---|---|
| Cholera - - - | 12 | Diarrhœa - - | 16 |
| Pleurisy - - - | 3 | Colic - - - | 2 |
| Acute Rheumatism - | 2 | Dysentery - - | 3 |
| Synochus - - | 3 | Gastrodynia - - | 7 |
| Gout - - - | 1 | Enterodynia - - | 3 |
| Apoplexy - - | 1 | Dyspepsia - - | 10 |
| Tic Douloureux - | 1 | Constipation - - | 3 |
| Hooping Cough - | 2 | Calculus - - - | 1 |
| Acute Diseases of Infants | 9 | Dysure - - - | 4 |

Lumbago

| | | | |
|---|---|---|---|
| Lumbago - - | 4 | Hydrocephalus - | 3 |
| Sciatica - - - | 2 | Ulcerated Sore Throat | 3 |
| Chronic Rheumatism | 3 | Gonorrhœa - - | 4 |
| Pleurodyne - - | 2 | Abortion - - | 2 |
| Cough and Dyspnœa | 10 | Leucorrhœa - - | 4 |
| Pulmonary Consumption | 6 | Prolapsus Uteri - | 1 |
| Scrophula - - | 2 | Scirrhus Uteri - - | 2 |
| Asthenia - - | 12 | Hysteria - - - | 2 |
| Paralysis - - | 2 | Amenorrhœa - - | 5 |
| Dropsy - - - | 2 | Menorrhagia - - | 2 |

During the last six weeks, complaints in the stomach and bowels have been very frequent and severe. Diarrhœa and cholera have affected numbers of people, not only in London, but in the surrounding country; and dysentery, bilious vomiting, and constipation with considerable pain; dyspepsia; and irregular action of the intestinal canal, have claimed the attention of the practitioner.

It is very usual to attribute the cause of these complaints to an indulgence in eating fruit. It cannot be denied that this is sometimes the case; but I believe much seldomer than is generally supposed. Disorders of the *primæ viæ* occur chiefly at the latter end of summer, and the beginning of autumn, and more especially when the season has been very warm, as was particularly the last. The vigour and strength of the body are then much impaired; the stomach and bowels are less able to perform the office of digestion; obstructions take place, which occasion inverted peristaltic motion, and the train of symptoms which usually accompany it; and besides all this, heat seems to have a direct influence upon the liver, which secretes more bile than is necessary for the system. Thus we may have all these complaints without any fruit being taken; and so far from its being prejudicial, it may be regarded as peculiarly beneficial at a period when there is so much tendency to vitiated secretion. Two or three of my patients eat fruit, moderately, the day after a severe attack of cholera, without any increase of their symptoms; and very few of them had indulged in fruit or vegetables, previous to their attack, which, in some instances, I had reason to think was occasioned by the too liberal use of wine and malt-liquor. It is well known that dysentery generally prevails in camps during the season when fruit is most plentiful; hence superficial observers have very frequently considered the complaint to be produced by excess in that article. Sir John Pringle, however, who had large experience experience upon this subject, has stated, that out of many hundred patients whom he attended in dysentery, he never once found it occasioned by eating fruit.

The cases of cholera which I attended, recovered; but I was sent for to see a young woman who died before I could reach the house; she had been ill three days before they thought proper to call in advice; though this complaint is always so distressing and alarming. It is very common for the poor, and even the middle rank of people, when first seized with any bowel-complaint, to swallow a glass or two of ardent spirits; if this is not effectual, they have recourse to a large dose of tincture of rhubarb; which ensures them a Doctor, and sometimes an Undertaker. SAMUEL FOTHERGILL.

*Southampton Street, Strand.*

